# Advancing Tomographic Volumetric Printing Via Oxygen Inhibition Control: Improved Accuracy and Large‐Volume Capability

**DOI:** 10.1002/adma.202508729

**Published:** 2025-09-12

**Authors:** Yujie Zhang, Katherine Houlahan, Daniel Webber, Nicolas Milliken, Kathleen L. Sampson, Hendrick W. de Haan, Hao Li, Robynne Vlaming, Liliana Gaburici, Antony Orth, Chantal Paquet

**Affiliations:** ^1^ National Research Council Canada Ottawa ON K1N 5A2 Canada; ^2^ Ontario Tech University Oshawa ON L1G 0C5 Canada

**Keywords:** large‐volume printing, (meth)acrylate photopolymerization, oxygen inhibition, print fidelity, tomographic volumetric additive manufacturing

## Abstract

Tomographic volumetric additive manufacturing (TVAM) is an emerging 3D printing technology capable of producing complex structures in seconds. However, achieving reliable prints using TVAM requires sufficient light penetration throughout the print volume, which often limits the photoinitiator (PI) concentration that can be used. In (meth)acrylate‐based photoresins, this constraint severely restricts achievable print size and quality due to oxygen inhibition. To address this challenge, a chemical strategy is demonstrated to control the oxygen inhibition period without compromising light penetration, using an amine, a thiol, and a phosphine additive as representative examples. Among these, N‐methyldiethanolamine (MDEA) emerged as the most promising candidate, effectively reacting with non‐reactive peroxy radicals to regenerate propagating radicals and sustain polymerization. Incorporating MDEA into a low‐PI photoresin enabled high‐resolution and large‐volume printing in a custom‐built TVAM system, achieving a root‐mean‐square surface deviation of 0.175 mm (≈2 pixels) and printable structure sizes up to 60 mm. These advances represent a 16‐fold increase in print volume relative to the previous TVAM demonstrations and enable high‐throughput fabrication of multiple complex parts without sacrificing print quality. This work establishes a scalable approach to overcoming oxygen inhibition in (meth)acrylate TVAM systems, unlocking new possibilities for large‐volume, high‐resolution additive manufacturing.

## Graphical abstract

Oxygen inhibition limits the printable volume and resolution in tomographic volumetric additive manufacturing using (meth)acrylate photoresins. This study presents a simple yet strategic formulation design using additives that regenerate active radicals from peroxyl radicals. Incorporating N‐methyldiethanolamine enables high‐resolution, large‐volume printing of complex structures up to 60 mm, achieving a 16‐fold increase in print volume without compromising print fidelity or speed.

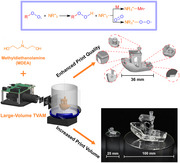

## 1 Introduction

**Figure 1:**
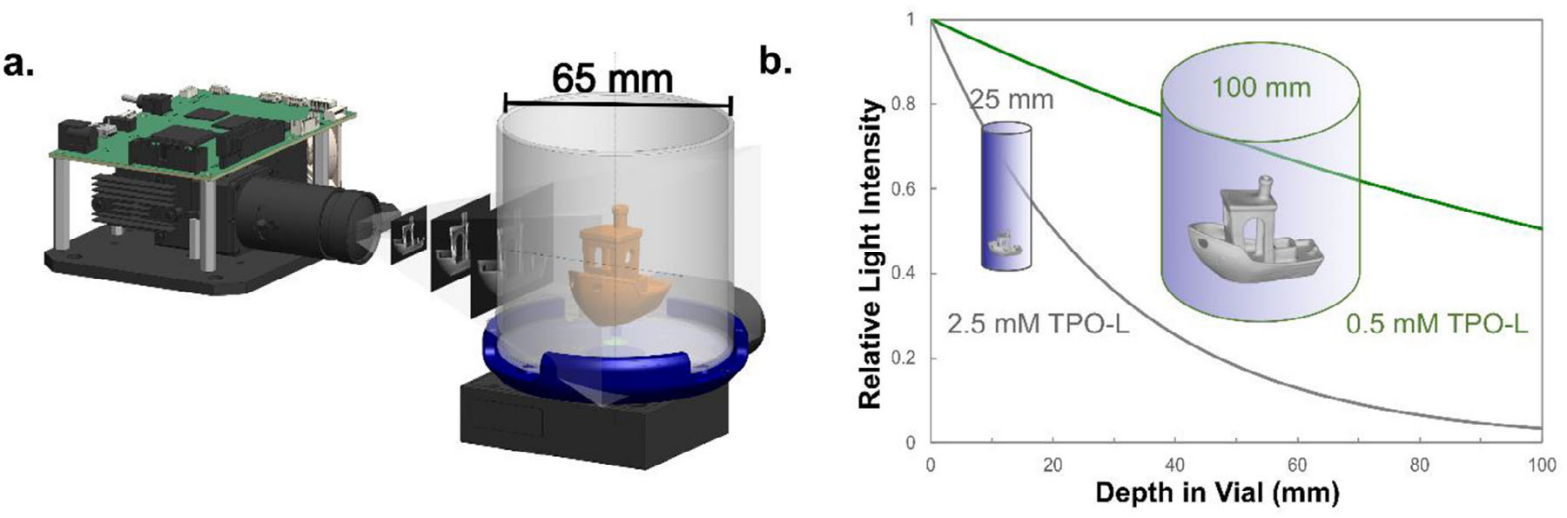
a) A large‐volume volumetric printer with a 65 mm vial diameter. b) Simulated light attenuation profiles in a vial containing photoresins with TPO‐L at concentrations of 0.5 mм (green) and 2.5 mм (gray), calculated using the Beer–Lambert law with an extinction coefficient of 137 м^−1^cm^−1^. The concentrations of 0.5 and 2.5 mм correspond approximately to the TPO‐L concentrations required for sufficient exposure across vial diameters of 100 and 25 mm in TVAM printing, respectively.

Tomographic volumetric additive manufacturing (TVAM) is emerging as a powerful light‐based 3D printing technology to rapidly fabricate complex objects.^[^ ^1^, ^2^, ^3^, ^4^ ^]^ Using a process most akin to computed tomography (CT) in reverse, light patterns computed using tomographic methods are projected through a rotating vial containing photoresin (**Figure** **1**a). Once the light dose absorbed by the photoresin exceeds the gelation threshold, the photoresin solidifies to form the desired 3D object. This volumetric approach to 3D printing eliminates staircasing artifacts, requires no support structures, and generates objects in a fraction of the time of conventional 3D printing methods. These benefits make TVAM an attractive high‐throughput manufacturing tool for rapid prototyping and fabricating optical components, engineered tissue, and other advanced materials.^[^ ^5^, ^6^, ^7^ ^]^

Unlike conventional layer‐based printing, TVAM exposes the entire print volume simultaneously with a spatially varied light dose, placing unique requirements on the photoresin formulation. First, the photoresin must exhibit a gelation threshold, ensuring that only regions receiving sufficient light dose (i.e., in‐part volumes) solidify, while out‐of‐part volumes remain liquid. 2,2,6,6‐Tetramethylpiperidine 1‐oxyl (TEMPO) is commonly added to thiol‐ene‐based photoresin to introduce the gelation threshold.^[^ ^8^, ^9^, ^10^, ^11^ ^]^ (Meth)acrylate‐based systems naturally meet this requirement due to oxygen inhibition, which prevents polymerization until sufficient photo‐radicals are generated to consume dissolved oxygen. Second, the photoresin must allow sufficient light to penetrate the entire print depth, forcing the use of extremely low photoinitiator (PI) concentrations.^[^ ^12^ ^]^

The necessity of employing low PI concentrations in TVAM leads to several significant challenges. Lower PI concentrations reduce the radical generation rate, resulting in slower polymerization rates. This increases overall print time and exacerbates printing defects due to part sedimentation. More importantly, oxygen inhibition and diffusion plays a central role in limiting both resolution and print fidelity in (meth)acrylate TVAM systems.^[^ ^1^, ^12^ ^]^ Due to the spatially varying light dose inherent to TVAM, in‐part regions receive significantly more light than out‐of‐part regions. This imbalance leads to non‐uniform oxygen consumption across the photoresin volume. Depletion of oxygen in the in‐part volumes will be replenished by the oxygen‐rich out‐of‐part volumes via diffusion. As a consequence, small print features on the scale of the oxygen diffusion length are difficult to form, as they experience prolonged inhibition periods and struggle to reach the gelation threshold within the standard exposure time. This results in incomplete curing, blurred boundaries, and ultimately degraded print fidelity. Although computational methods can mitigate these printing artifacts,^[^ ^1^, ^13^ ^]^ limitations in print quality and achievable sizes persist.

Conversely, increasing PI concentrations to improve print quality leads to excessive light attenuation, reducing penetration depth and causing underexposure at the center of the printed parts.^[^ ^12^ ^]^ This underexposure compromises print fidelity, often resulting in poor accuracy or print failure. Consequently, (meth)acrylate photoresins must strike a delicate balance: PI concentrations must be high enough to ensure sufficient radical generation for oxygen depletion and polymerization, yet low enough to permit sufficient light penetration through the entire print volume (Figure 1b). We have previously shown that as the diameter of the print volume increases, the required PI concentration must decrease to maintain light penetration, eventually reaching a threshold where radical generation becomes insufficient to support polymerization.^[^ ^12^ ^]^ For example, we demonstrated that the minimum penetration depth that can yield a good quality 3DBenchy (mean root‐mean‐squared (RMS) surface deviation of 0.214 mm) using ethyl (2,4,6‐trimethylbenzoyl) phenylphosphinate (TPO‐L) as the PI in the diurethane dimethacrylate (DUDMA) photoresin is ≈1.3 cm.^[^ ^12^, ^14^ ^]^ Thus, the requirement for low PI concentrations fundamentally constrains both the maximum print volume and the minimum achievable feature size in (meth)acrylate TVAM systems.

Recent hardware innovations, such as tomographic projections along a helical path in tall cylindrical volumes and roll‐to‐roll platforms, have extended print length in one or two dimensions.^[^ ^15^, ^16^, ^17^ ^]^ Although these approaches increase the length of printable geometries in one or two dimensions, light attenuation still limits the maximum cross‐sectional area of the prints. In (meth)acrylate systems, this challenge is compounded by the excessive loss of photogenerated radicals due to oxygen inhibition. Rather than turning to oxygen‐tolerant chemistries such as thiol‐ene, which involve distinct polymerization mechanisms and limitations, we propose a strategy that operates within the (meth)acrylate framework. Inspired by previously established peroxyl radical chemistries,^[^ ^10^, ^18^, ^19^ ^]^ we incorporate additives that regenerate terminated peroxyl radicals, offering a practical means to significantly increase print volume and enhance overall print quality.

Here, we demonstrate that additives, namely amines, thiols, and phosphines, can regenerate reactive radicals from peroxyl species formed during oxygen inhibition. These regenerated radicals either initiate polymerization directly or further react with oxygen, effectively amplifying the number of functional propagating radicals per photon absorbed.^[^ ^18^, ^19^, ^20^ ^]^ This allows for the use of even lower PI concentrations without sacrificing polymerization efficiency in (meth)acrylate TVAM systems. The resulting formulations ensure sufficient light penetration for large‐volume printing while also mitigating common low‐PI issues, such as reduced print resolution. Through simple yet strategic formulation design, this work advances the scalability and robustness of (meth)acrylate TVAM systems, addressing a key bottleneck and expanding its practical application space.

## 2 Results and Discussion

### 2.1 Evaluation of Oxygen Inhibition Reduction Chemicals

**Figure 2:**
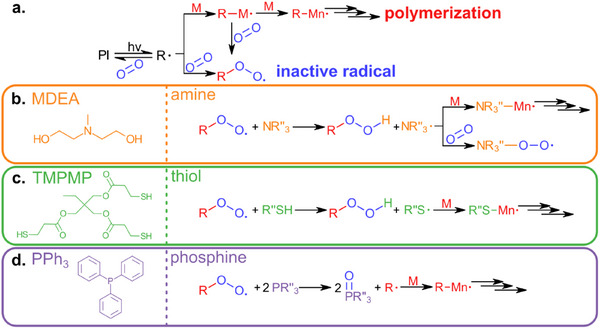
Comparison of different additives. a) Photopolymerization reaction mechanism showing the inhibition with oxygen to form an inactive radical, which is dominated over the polymerization pathway until most of the oxygen is consumed. b) Structure of MDEA additive and mechanism of reducing oxygen inhibition via hydrogen donation with an amine additive. c) Structure of TMPMP additive and mechanism of reducing oxygen inhibition via hydrogen donation with a thiol additive. d) Structure of PPh_3_ additive and mechanism of reducing oxygen inhibition via reducing agent with a phosphine additive.

In typical (meth)acrylate photopolymerization, oxygen acts as an inhibitor by reacting with active polymer radicals (R• or R‐M•) to form peroxyl radicals (ROO•) (**Figure** **2**a; Scheme S1, Supporting Information), which have very limited reactivity toward propagating the polymer chain.^[^ ^18^, ^19^, ^21^ ^]^ Polymerization is thus suppressed until the local oxygen concentration is sufficiently depleted, allowing (meth)acrylates to compete effectively for R• or R–M• radicals. This delay in (meth)acrylate polymerization underpins the threshold behavior required for successful TVAM printing. Therefore, the efficient use of photogenerated radicals is critical in (meth)acrylate systems, where PI concentrations must be kept low to enable sufficient light penetration. Compounds capable of regenerating reactive radicals from peroxyl radicals offer a strategy to mitigate the effects of oxygen inhibition in (meth)acrylate photoresins while preserving the necessary gelation threshold. Here, we evaluate three groups of additives, namely amines, thiols, and phosphines, for their ability to regenerate reactive radicals and reduce oxygen inhibition in (meth)acrylate TVAM systems.

Amines and thiols mitigate oxygen inhibition by donating hydrogen atoms to peroxyl radicals (ROO•), generating aminoalkyl radicals (Figure 2b) and thiyl radicals (Figure 2c), respectively. The aminoalkyl and thiyl radicals can subsequently react with (meth)acrylate monomers to continue polymerization. Additionally, aminoalkyl radicals can react with oxygen to form aminoalkyl peroxyl radicals, further consuming oxygen in the system.^[^ ^18^, ^20^, ^21^ ^]^ Phosphines act as reducing agents that interact with peroxyl radicals, regenerating carbon‐centered propagating radicals (R•) (Figure 2d).^[^ ^18^, ^21^, ^22^ ^]^ Collectively, these additives provide pathways to regenerate active carbon‐centered radicals, which can react either with (meth)acrylate monomers or oxygen, ultimately reducing oxygen depletion time and sustaining radical availability even at reduced PI concentrations.

The three additives screened in this study were N‐methyldiethanolamine (MDEA), trimethylolpropane tris(3‐mercaptopropionate) (TMPMP), and triphenylphosphine (PPh_3_). Their performance was evaluated by the time required to deplete oxygen (via photo‐rheology), absorption at the projector wavelength (405 nm), printability, and photoresin stability in DUDMA photoresin. Additive concentrations ranging from 0.88 to 238.77 mм were tested to evaluate their effectiveness in mitigating oxygen inhibition.

**Figure 3:**
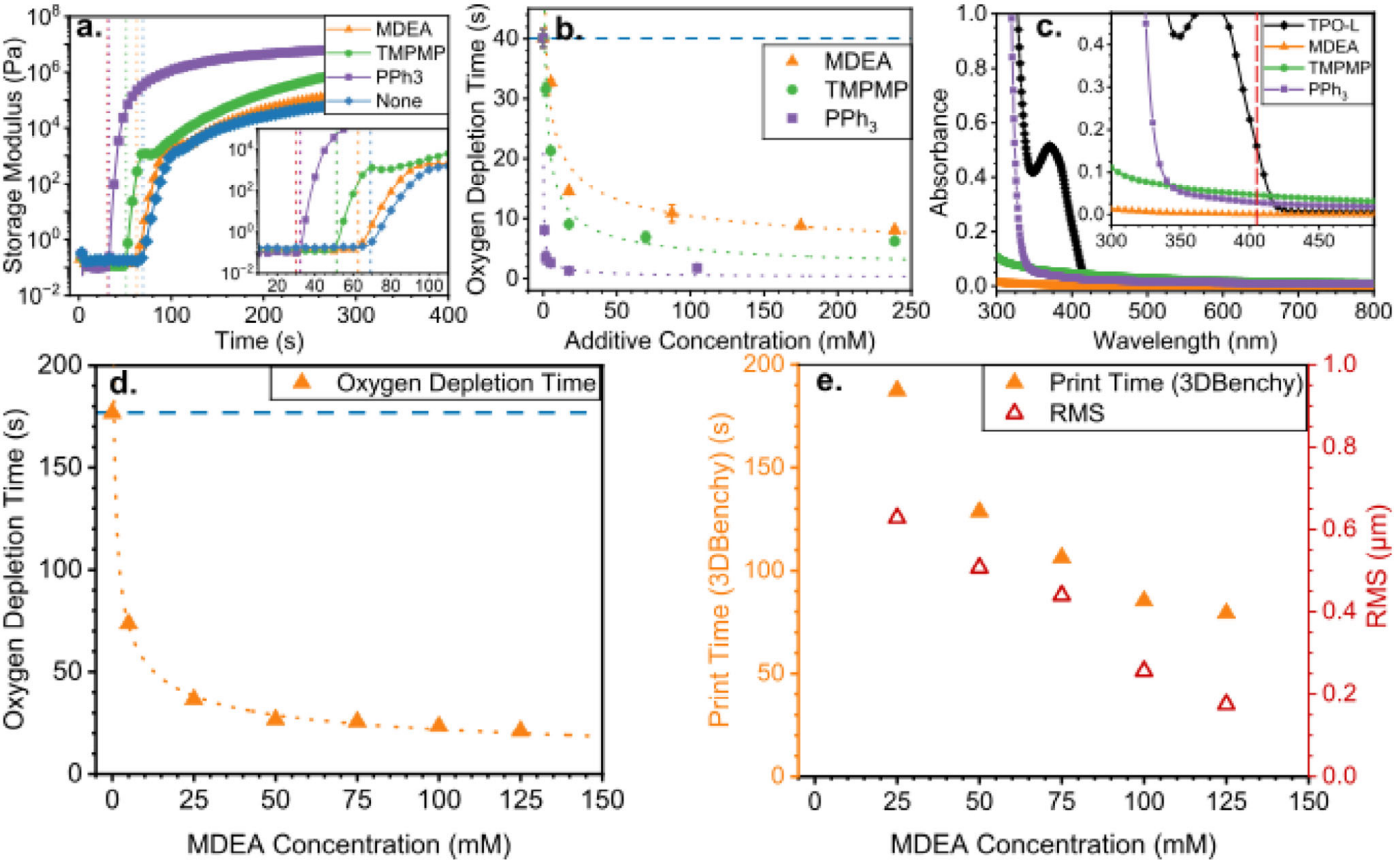
a) Photo‐rheology experiments with 5.25 mм MDEA (orange triangle), TMPMP (green circle), and PPh_3_ (purple square) additives compared to no additive (blue triangle) in the DUDMA photoresin with 1.75 mм TPO‐L showing the change in storage modulus as a function of time. The dotted red line corresponds to the start of 405 nm light exposure with 5 mW cm^−2^ light intensity at 30 s. The dotted orange, green, purple, and blue vertical lines highlight the onset of polymerization and oxygen depletion times for the MDEA, TMPMP, PPh_3_ additives, and no additive, respectively. b) Oxygen depletion time measured by photo‐rheology at various additive concentrations with MDEA (orange triangle), TMPMP (green circle), and PPh_3_ (purple square) additives with the DUDMA photoresin at 1.75 mм TPO‐L. Trend lines were fit to a power curve (Table S1, Supporting Information). The horizontal dashed blue line corresponds to the oxygen depletion time of the photoresin without additive. c) UV–vis absorption of 0.07 wt.% TPO‐L, 2.66 wt.% MDEA, 0.50 wt.% TMPMP, and 0.50 wt.% PPh_3_ in ethanol. The inset is of the region around the wavelength of the TVAM printer at 405 nm (vertical dashed red line). d) Oxygen depletion time measured by photo‐rheology and plotted relative to MDEA concentrations using D8P2 photoresin with 0.5 mм TPO‐L. Trend lines were fit to a power curve (details in Supporting Information). The horizontal dashed blue line corresponds to the oxygen depletion time of the photoresin without MDEA additive. e) Printing time for a 36 mm length 3DBenchy, alongside the RMS deviation of the computed signed‐distance functions (SDF) of the printed 3DBenchy across various MDEA concentrations using D8P2 photoresin with 0.5 mм TPO‐L.

Photo‐rheology was used to assess the ability of additives to reduce the oxygen depletion time following our previously described approach.^[^ ^12^ ^]^ Exemplary storage modulus curves of photoresins containing MDEA, TMPMP, and PPh_3_, shown in **Figure** **3**a, reveal that the onset of polymerization occurs more rapidly in photoresins with additives compared to the additive‐free control. The effect of increasing additive concentration is shown in Figure 3b, where the oxygen depletion time decreases nonlinearly with concentration. Beyond a certain threshold, further increases in additive concentration yield diminishing returns in reducing the oxygen inhibition period, indicating a shift from a diffusion‐limited regime at lower concentrations to a reaction‐limited regime at higher concentrations, and suggesting a saturation limit for each additive's ability to suppress oxygen inhibition. Among the three, PPh_3_ was the most effective in reducing the oxygen depletion time, followed by TMPMP and then MDEA, consistent with previous findings by Husár et al.^[^ ^21^ ^]^ Overall, the oxygen depletion time was reduced to 20.0%, 15.5%, and 3.4% of the value observed in the additive‐free photoresin for MDEA, TMPMP, and PPh_3_, respectively, at the highest concentrations tested. Importantly, the inhibition period must be preserved upon additive incorporation to maintain the gelation threshold essential for successful TVAM printing.^[^ ^3^ ^]^

To avoid interfering with the TVAM printing process, it is critical that additives exhibit minimal absorption at the printing wavelength of 405 nm. UV–vis absorption measurements (Figure 3c) show that, compared to TPO‐L, the additional absorbance contributions from MDEA, TMPMP, and PPh_3_ were less than 1%, ≈29%, and ≈18%, respectively. Thus, MDEA is expected to have the least effect on light penetration and dose distribution during TVAM printing. The additional absorbance should be considered when computing the tomographic projections to achieve more accurate print fidelity and may ultimately limit the printable volume in formulations with high additive absorbance.

The printability of photoresins containing different concentrations of each additive was evaluated by fabricating dog bone structures (Figure S1, Supporting Information) in 25 mm diameter vials. Photoresins formulated with MDEA generally produced high‐quality prints across the tested concentration range (Figure S2, Supporting Information). However, at the highest concentration tested (238.77 mм), high print speed led to overcuring and a decline in print quality, as it became difficult to terminate the print at the optimal time. For TMPMP, successful prints were achieved at concentrations of 1.75, 5.25, and 17.50 mм. At higher concentrations (69.74 and 238.77 mм), printability declined, with frequent overprinting, degraded surface finish (Figure S3, Supporting Information), and unintended curing in out‐of‐part region (Figure S4, Supporting Information). These issues are attributed to the shortened oxygen inhibition period and a reduced gelation threshold, which compromised spatial control and print resolution. In the case of PPh_3_, the usable concentration range was narrower. While successful prints were achieved at 0.88 mм, concentrations at or above 1.75 mм led to significant overcuring and surface deterioration (Figure S5, Supporting Information). This behavior is attributed to an extremely short inhibition period, making it difficult to precisely control the exposure time and terminate the print before overcuring occurred. The specific requirements for the inhibition period depend on factors such as the geometry, spatial distribution of light dose, photoresin properties, and whether the printing system uses manual or automated exposure termination.^[^ ^13^, ^23^ ^]^

In addition to printability, photoresin stability and reusability were considered. It is well known that photoresins with both phosphines and thiols have lower storage stability compared with MDEA, especially under ambient conditions and at higher additive concentrations; in particular, photoresins containing PPh_3_ can lose its stability within 24 h.^[^ ^18^, ^21^ ^]^ This instability, combined with a narrow printing window, also makes it difficult to reuse TMPMP‐ and PPh_3_‐containing photoresins. In addition, PPh_3_ was not readily soluble in the photoresin, and a small amount of toluene was required for incorporation, which could impact printability at higher concentrations.

Considering the impact on oxygen inhibition period, light absorption, print quality, photoresin stability and reusability, and ease of formulation, MDEA was selected as the most suitable additive. Although it was the least effective at shortening the oxygen inhibition time (80.0% reduction at the highest concentration), it consistently produced high‐fidelity prints with stable photoresin behavior and minimal impact on light penetration. Its broader printing window allowed for more reliable identification of the optimal print completion time, which is critical for achieving successful TVAM printing of high‐quality parts. While TMPMP is a viable alternative, its effective concentration range is narrower and requires careful control to maintain the gelation threshold. Overall, MDEA provides the most balanced combination of shortening the oxygen inhibition period, increasing print quality, and preserving photoresin shelf‐life, making it well‐suited for large‐volume TVAM applications with (meth)acrylate photoresins.

### 2.2 Large‐Volume TVAM Printing

With MDEA identified as a promising additive to reduce oxygen inhibition period in (meth)acrylate systems, we investigated its effect on large‐volume TVAM. A range of MDEA concentrations (0–125 mм) were tested in the photoresin containing 80 wt.% DUDMA and 20 wt.% poly(ethylene glycol) diacrylate (PEGDA) with 0.5 mм TPO‐L as the PI, referred as “D8P2 photoresin” below. The TPO‐L concentration was selected to ensure sufficient penetration depth for printing vials up to 100 mm in diameter based on penetration depth calculation from our previous work.^[^ ^12^ ^]^ The oxygen depletion time for each formulation was first characterized using photo‐rheology, revealing that increasing MDEA concentration progressively reduced oxygen depletion time (Figure 3d) in a similar fashion as above with 1.75 mм TPO‐L (Figure 3a,b). Specifically, adding 5 mм MDEA significantly decreased the depletion time from 176.8 to 73.6 s. However, beyond 50 mм, further reductions were marginal. This concentration corresponds to an additive‐to‐PI molar ratio of ≈100:1, consistent with our preliminary screening results, where increasing the MDEA concentration beyond a 100:1 ratio (175 mм) produced negligible additional improvements in oxygen inhibition. Thus, MDEA effectively decreases oxygen depletion time, though the optimal concentration may depend on the PI (TPO‐L) concentration used.

**Figure 4:**
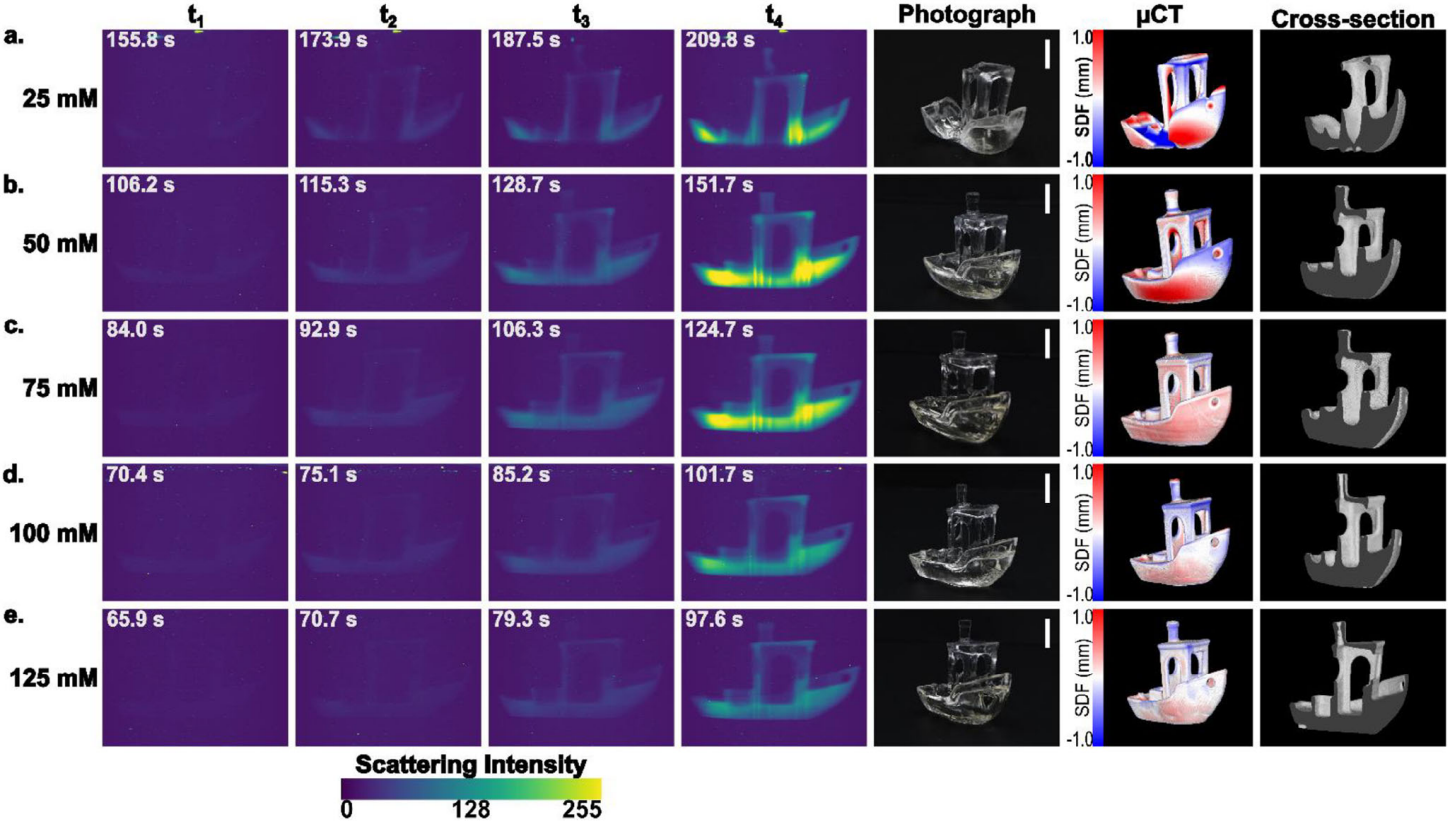
Effect of MDEA concentration on TVAM printing. Samples were printed using D8P2 photoresin at MDEA concentrations of a) 25 mм, b) 50 mм, c) 75 mм, d) 100 mм, and e) 125 mм. Each panel includes optical scatter imaging at key stages of the 3DBenchy printing process (t₁: hull formation, t_2_: cabin wall formation, t_3_: chimney formation and print termination, and t_4_: 18 s after print was stopped), the photograph of the printed part, the SDF comparing the µCT‐derived isosurface to the target design, and a representative cross‐section. Scale bars: 5 mm.

To assess print quality across different MDEA concentrations, a 3DBenchy model with a length of 36 mm was printed inside a 65 mm vial and the process was monitored using real‐time optical scattering tomographic imaging (OST) as previously described.^[^ ^14^, ^24^ ^]^ The projection was computed using 3D ray tracing and a proportional‐integral feedback loop was implemented.^[^ ^4^, ^25^ ^]^ This projection resulted in an average non‐zero grayscale value of 13% of the projector maximum, corresponding to a light intensity of 3.2 mW cm^−2^ at the vial center. **Figure** **4** presents OST images at four key stages of printing: the initial appearance of the hull, the formation of the cabin walls, the formation of the chimney just before the print was stopped, and an image taken two rotations after stopping the print (18 s later). In addition to OST images, micro‐computed tomography (µCT) was used to reconstruct 3D models of the printed parts and analyze their geometric accuracy (Figure 4; Table S2, Supporting Information). The µCT reconstructions were further compared to the reference geometry by calculating signed‐distance function (SDF) deviations from the target, and the RMS surface deviations were reported (Figure 3e).

Prints were unsuccessful below 25 mм MDEA, as insufficient polymerization prevented the formation of stable cabin walls to support the roof due to the significant impact of oxygen inhibition. At 25 mм, all key features of the 3DBenchy, including the hull, cabin walls, and chimney, were formed during printing; however, the chimney fractured during post‐processing due to incomplete polymerization. At concentrations above 25 mм, successful prints were obtained with all key features intact. At 100 mм MDEA, fine details such as the chimney holes became clearly distinguishable, while at 125 mм MDEA, the blind hole was fully resolved (Table S3, Supporting Information). The RMS deviation between printed 3DBenchys and the reference geometry decreased with increasing MDEA concentration, indicating improved print fidelity, as shown in Figure 4 and Table S2 (Supporting Information). The 3DBenchy printed with 125 mм MDEA exhibited high resolution with an RMS deviation of 0.175 mm (≈2 pixels) (Figure 4e).

Increasing MDEA concentration also led to a reduction in total printing time, decreasing from 187.5 s at 25 mм to 79.3 s at 125 mм (Figure 4), as expected from photo‐rheology experiments. The time difference between the formation of the hull (a large feature) and the chimney (a fine feature) also decreased, from 31.7 s at 25 mм to 13.4 s at 125 mм, indicating a more uniform print time for different feature sizes. This effect explains the observed over‐curing of the hull and under‐curing of the finer features at lower MDEA concentrations, as confirmed by µCT images. Analysis of OST imaging further supports this observation, showing that at higher MDEA concentrations, the scattering intensity difference between the hull and the chimney decreased, demonstrating improved uniformity in print time (Figure 4). Thus, MDEA functions as a powerful chemical tool to mitigate a key limitation in (meth)acrylate TVAM systems: oxygen inhibition and diffusion causing feature‐size‐dependent variation in print speed. By accelerating oxygen depletion and increasing effective radical availability, MDEA reduces print time, enables larger print volumes, and improves the resolution of fine features.

**Figure 5:**
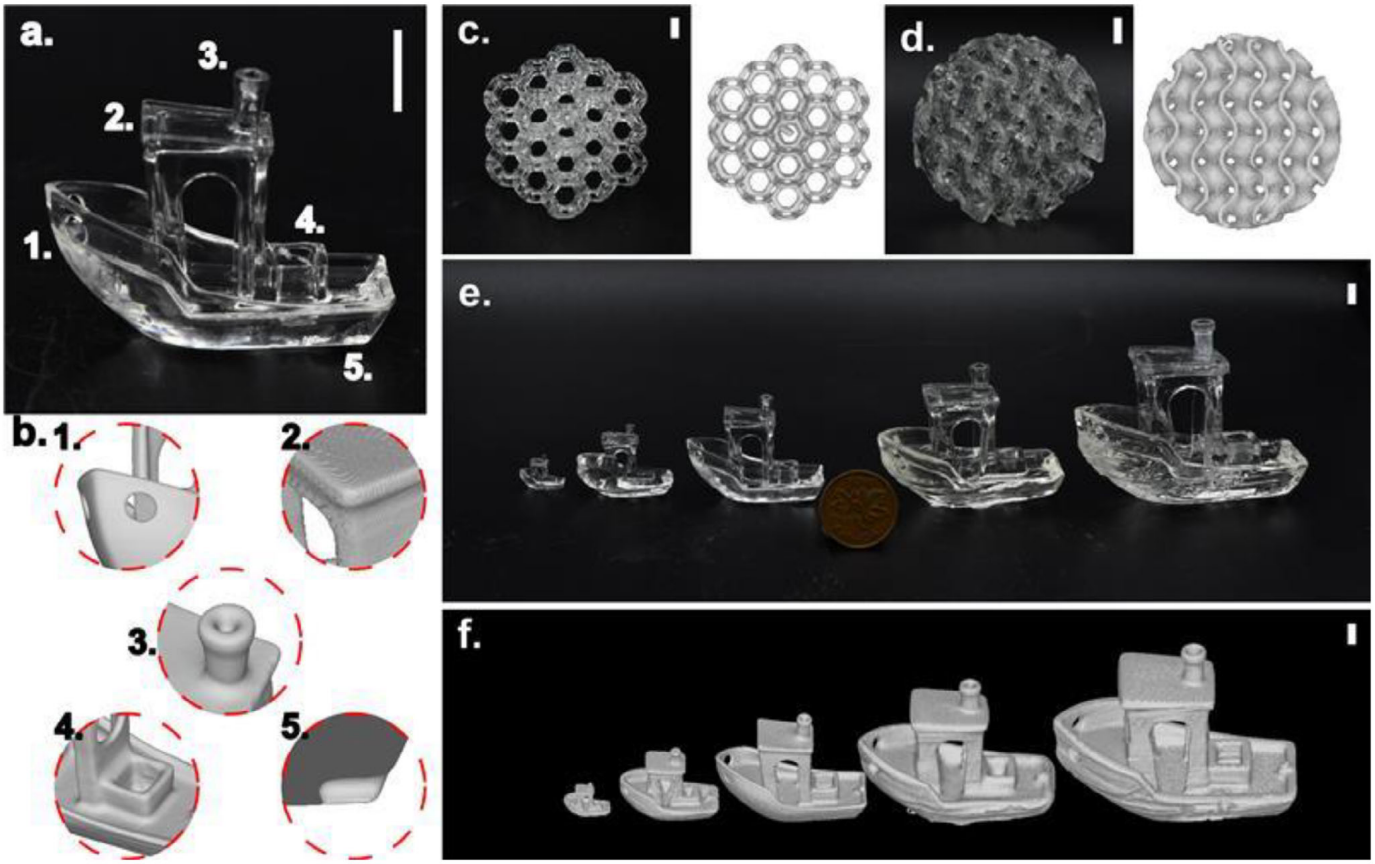
Demonstration of print versatility and increased achievable print size using D8P2 photoresin with 0.5 mм TPO‐L and 125 mм MDEA. a) 3DBenchy (36 mm length) printed with PIHE optimization. b) µCT reconstruction of the print shown in (a), highlighting key structural features. c) Cubic Kelvin lattice with a side length of 30.0 mm and strut thickness of 1.25 mm, shown alongside its µCT reconstruction. d) Cylindrical gyroid lattice with a diameter of 40.0 mm, height of 20.0 mm, and wall thickness of 1.5 mm, shown alongside its µCT reconstruction. e) Series of 3DBenchy prints with lengths of 12.0, 24.0, 36.0, 48.0, and 60.0 mm, illustrating the scalability of the process. f) µCT reconstructions of the 3DBenchy shown in (e). Scale bars: 5 mm.

Building on our previous work showing that higher light intensities improve both print speed and fidelity,^[^ ^12^ ^]^ all subsequent projections were optimized using proportional‐integral histogram equalization (PIHE), which increased the light intensity at the vial center to 7.8 mW cm^−2^. This optimization was further validated by printing a 36 mm 3DBenchy using 125 mм MDEA with PIHE‐adjusted projections, resulting in a reduced printing time of 43.7 s and enhanced fine‐feature resolution (**Figure** **5**a,b; Video S1, Supporting Information).

Once the optimal MDEA concentration was identified along with the projection optimization method, additional geometries were printed to evaluate the capability to fabricate complex structures. A cubic Kelvin lattice measuring 30.0 mm in length with a strut thickness of 1.25 mm (Figure 5c), as well as cylindrical gyroid lattice with a 40.0 mm diameter, 20.0 mm height, and 1.5 mm wall thickness (Figure 5d), were successfully printed, both exhibiting good print quality.

The sufficient light penetration of this formulation was further validated by printing within a 100 mm diameter vial, representing a 16‐fold increase in printable volume compared to additive‐free photoresins.^[^ ^12^ ^]^ A series of prints of varying sizes, including 3DBenchy (Figure 5e,f) and Bulbasaur models (Figure S6a,b, Supporting Information), were fabricated, with 3DBenchys ranging from 12.0 to 60.0 mm in length (corresponding to heights of 9.6 to 48.0 mm) and Bulbasaur models ranging from 16.0 to 60.3 mm in length (with heights from 13.0 to 49.0 mm).^[^ ^14^, ^26^ ^]^ The printing times varied from 35.2 to 58.2 s (Table S4, Supporting Information). Print quality remained high across different sizes; however, for the 3DBenchy models at 48 and 60 mm in length, over‐curing of the hull (a larger feature) was observed despite identical photoresin formulation and printing conditions. This effect is likely attributed to heat accumulation during polymerization due to its exothermic nature (Figure S7, Supporting Information).^[^ ^27^ ^]^ In geometries such as 3DBenchy, larger features (the hull) and smaller features (the cabin walls and chimney) are spatially separated in the print volume. This spatial separation leads to uneven heat distribution, which may alter local curing rates and trigger thermal convection within the photoresin, and thus, result in inconsistent print quality within the geometry. In contrast, the Bulbasaur model exhibited more uniform heat distribution, leading to greater consistency in print quality despite the size increase. While heat buildup can accelerate polymerization and reduce overall printing time, it may also introduce geometry‐dependent variations in print quality, highlighting the need to carefully manage thermal effects when scaling up TVAM prints. Infill or partially hollow geometries can be incorporated in place of fully solid structures to help mitigate excessive heat accumulation when printing large TVAM prints.

**Figure 6:**
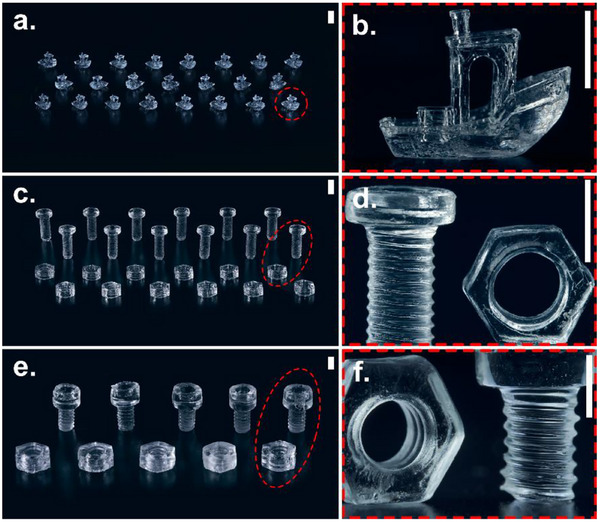
Simultaneous fabrication of multiple objects using D8P2 photoresin containing 0.5 mм TPO‐L and 125 mм MDEA. a) Array of 23 identical 3DBenchy prints (12 mm length) produced in a single print run. b) Representative 3DBenchy print from (a). c) Twelve sets of M5 screws and nuts printed simultaneously. d) Representative M5 screw and nut from (c). e) Five sets of ¼‐20 cap screws and corresponding nuts fabricated in a single batch. f) Representative ¼‐20 cap screw and nut from (e). Scale bars: 5 mm.

Beyond demonstrating increased print size, simultaneous fabrication of multiple objects was used to showcase the enhanced throughput and assess the system's ability to maintain uniform print quality across the entire build volume. To demonstrate this capability, 23 3DBenchys (12 mm in length, **Figure** **6a**,b), 12 sets of M5 screws and nuts (Figure 6c,d), and 5 sets of ¼‐20 cap screws and nuts (Figure 6e,f; Video S2, Supporting Information) were printed within a 65 mm vial, with the printing time of 54.1, 47.5 and 41.7 s, respectively. The printed 3DBenchys had an average RMS deviation of 0.234 ± 0.039 mm, and the ¼‐20 nuts were successfully threaded onto the corresponding cap screws, indicating good geometric accuracy. To achieve consistent quality in multi‐object prints, it is necessary to consider both the spacing between prints and their relative orientation. Based on previous studies, a minimum inter‐print distance of 2 mm is recommended to ensure that oxygen inhibition effects remain consistent across prints, thereby preventing variations in polymerization and print quality.^[^ ^12^ ^]^ Detailed designs for multi‐object printing are provided in the Supporting Information (Videos S3–S5, Supporting Information).

Scaling to even larger print volumes introduces additional formulation and system‐level constraints. From a materials perspective, sufficient radical generation is required to reach the gelation threshold across the full volume. In this work, we introduced additives that regenerate active radicals from the peroxyl radicals formed during oxygen inhibition, enabling the use of lower PI concentrations while maintaining reactivity. However, achieving high resolution in larger volumes imposes further limitations: oxygen diffusion must be mitigated to prevent blurring of fine features, which constrains PI concentration, additive concentration, and photoresin viscosity. Beyond formulation, hardware and computational factors also impose limitations. The projector's light intensity, optical resolution, and throw ratio determine the achievable voxel size and dose uniformity. The rotational stage must support heavier photoresin volumes and glass vials without introducing mechanical instability. Commercial cylindrical vials with sufficient optical quality and tight tolerances (typically 50–100 µm) become increasingly difficult to source at larger sizes. Scaling beyond ≈10 cm would likely require a custom‐fabricated, reusable print chamber with high optical clarity and dimensional precision. This would also necessitate changes in photoresin handling, such as fluidic systems to pump photoresin in and out, introducing additional complexity and cost. Moreover, larger print volumes require significantly more computational resources for light dose calculations, often necessitating downsampling which may degrade resolution. Together, these interdependent tradeoffs across photopolymerization chemistry, optics, mechanics, and computation define the practical boundaries of scalable TVAM printing.

### 2.3 Comparison to Commercial 3D Printers

Our formulation has significantly expanded the print volume of TVAM printers, enhancing their competitiveness with commercial 3D printing technologies. To assess key performance characteristics, a 36 mm 3DBenchy was printed using stereolithography (SLA), digital light processing (DLP), and fused deposition modeling (FDM) and compared in terms of voxel size, RMS deviation from the design geometry, print time, print rate, and voxel throughput (**Table** **1**; Figure S8, Supporting Information).

**Table 1:** Comparison of key characteristics between TVAM and other commercial 3D printing technologies when printing a 36 mm 3DBenchy.

Technique	Materials	Voxel volume [mm^3^]	RMS [mm]	Average print time [s]	Average print speed^a)^ [mm^3^ s^−1^]	Average voxel throughput [voxel s^−1^]
Large‐Volume TVAM [65 mm Vial]	D8P2 with 0.5 mм TPO‐L and 125 mм MDEA	6.25 × 10^−4^ ^b)^	0.229 ± 0.046	62	54.2	86673.7
SLA	Formlabs Clear Resin V5	2.50 × 10^−4^	0.126 ± 0.013	2865	1.2	4689.3
DLP	Formlabs Clear Resin V4	2.03 × 10^−4^	0.135 ± 0.018	5488	0.6	3022.3
FDM	Polyethylene Terephthalate Glycol (PETG)	3.20 × 10^−2^	0.235 ± 0.009	2072	1.6	50.7

^a)^ Print speed calculated as the volume of the 36 mm 3DBenchy divided by the corresponding print time;

^b)^ Theoretical voxel size at the vial center.

Compared to other vat polymerization‐based printers, our large TVAM system has a voxel size approximately two to three times larger, yet achieves a comparable RMS deviation, demonstrating high geometric accuracy. It is worth noting that further improvements in print quality can be achieved through hardware advancements and printer design, as we previously demonstrated.^[^ ^4^, ^12^ ^]^ A key advantage of TVAM is its significantly faster print speed. The 36 mm 3DBenchy was printed in ≈62 s using TVAM, whereas printing the same model required ≈46 times longer using an SLA‐based Formlabs Form 4 and ≈89 times longer using DLP. Compared to FDM, TVAM not only achieves similar print quality but also significantly reduces print time. These results highlight the importance of our optimized formulation, which enables large volume printing in TVAM. Without this advancement, TVAM printing has been restricted to small‐scale prints (<15 mm in length), despite its high speed. By overcoming previous volume limitations, this work positions TVAM as a viable alternative to existing 3D printing technologies, offering a unique combination of high print fidelity and rapid fabrication.

To further contextualize the performance of our large‐volume TVAM platform, we include a quantitative comparison with other high‐throughput 3D printing technologies (Table S5, Supporting Information), which were not available for direct benchmarking. Multiphoton polymerization (MPP) systems achieve sub‐micron resolution but are limited by low volumetric print rate and small build volumes, making them less practical for mesoscale fabrication.^[^ ^28^, ^29^ ^]^ Emerging techniques such as Xolography and Dynamic Interface Printing (DIP) demonstrate improved throughput but come with trade‐offs.^[^ ^30^, ^31^ ^]^ Xolography, for example, requires photoresins with high viscosity to maintain light‐sheet stability and is further constrained by alignment complexity.^[^ ^30^ ^]^ DIP achieves high voxel throughput but is limited by stringent photoresin requirements and design flexibility, particularly for overprinting and multi‐material fabrication.^[^ ^31^ ^]^ In contrast, our large‐volume TVAM system accommodates a broader range of photoresin viscosities and complex geometries, delivering multi‐centimeter‐scale prints with a print rate of 6.0 × 10^3^ mm^3^ s^−1^ (Table S5, Supporting Information) using a 100 mm diameter vial. This positions TVAM as a scalable and flexible platform for high‐speed mesoscale additive manufacturing.

## 3 Conclusion

In this study, we address one of the fundamental limitations of TVAM for (meth)acrylate systems, its restricted printable volume, by developing a chemical strategy to suppress oxygen inhibition without compromising light penetration. Through systematic screening of three additive classes, namely an amine (MDEA), a thiol (TMPMP), and a phosphine (PPh_3_), we identified MDEA as the most effective and robust additive for enabling large‐volume printing in oxygen‐sensitive (meth)acrylate photoresins. Using a custom‐built TVAM system with a 65 mm vial, we identified 125 mм as the optimal MDEA concentration for D8P2 photoresin, yielding high‐fidelity prints with feature resolution of ≈2 pixels.

We successfully fabricated high‐quality complex structures up to 60 mm in length with a print rate of 6.0 × 10^3^ mm^3^ s^−1^ using a 100 mm diameter vial, representing a 16‐fold increase in print volume compared to our previously reported systems. We further demonstrated high‐throughput potential by printing multiple parts simultaneously, highlighting the productivity gains of the MDEA‐modified photoresin and the system's ability to maintain uniform print quality across the entire build volume. To benchmark performance, a 36 mm‐long 3DBenchy was fabricated and compared to parts produced by commercial SLA, DLP, and FDM systems. The resulting TVAM print exhibited comparable fidelity while printing significantly faster than conventional technologies.

While interdependent tradeoffs across photopolymerization chemistry, optics, mechanics, and computation define the practical limits of scalability, this work establishes a robust and scalable framework for high‐resolution, large‐volume TVAM printing in (meth)acrylate systems by overcoming the oxygen‐inhibition barrier while maintaining high light penetration.

## 4 Experimental Section

### Photoresin Preparation

Two distinct photoresin formulations were prepared for this study.

For the preliminary tests using a 25 mm vial in the TVAM printer, DUDMA (Esstech Inc.) was combined with TPO‐L (Oakwood Chemical) at a concentration of 1.75 mм. This formulation was referred to as the “DUDMA photoresin”. In these tests, three additives were investigated to reduce oxygen inhibition: MDEA, TMPMP, and PPh_3_. All additives were procured from Sigma–Aldrich and introduced at various concentrations between 0.88 and 238.77 mм, corresponding to molar ratios of additive:PI between 0.5:1 and 136.43:1. All chemicals were used as received and thoroughly mixed using an ARE 310 Thinky planetary mixer. Due to the limited solubility of PPh_3_ in DUDMA, it was first dissolved in toluene at a concentration of 1.76 м before being incorporated into the photoresin. The prepared photoresins were stored in refrigerated conditions and kept protected from light until use. Before printing, the photoresins were transferred to a glass vial and allowed to settle until they reached room temperature and all visible bubbles dissipated.

For the large volume printer experiments, the photoresin formulation was modified by blending DUDMA with PEGDA (Mn = 700 g mol^−1^, Sigma–Aldrich) in an 80:20 weight ratio. A PI concentration of 0.5 mм TPO‐L was employed to ensure sufficient light penetration for the largest printing vial (99.6 mm diameter). This formulation was referred to as the “D8P2 photoresin”. MDEA was used as the oxygen inhibition reduction agent, with its concentration varied across experiments from 0 to 125 mм. The photoresin was mixed with an overhead mixer for 30 min, after which similar preparation procedures as stated above were followed.

Refractive index measurements of all photoresins were performed using a Schmidt‐Haensch ATR‐BR refractometer, and the detailed values are reported in Table S6 (Supporting Information).

### Absorbance and Penetration Depth Measurements

Absorbance measurements for TPO‐L, PPh_3_, TMPMP, and MDEA were performed in ethanol at selected concentrations using a Cary 5000 UV–vis–NIR Spectrophotometer between 300 to 800 nm. Additionally, the recent publication details the penetration depth of DUDMA photoresins across various TPO‐L concentrations.^[^ ^12^ ^]^

### Photo‐Rheology

Photo‐rheology measurements were carried out using a TA Instruments Discovery HR20 equipped with a UV light guide accessory for determining oxygen depletion time, as previously described.^[^ ^12^ ^]^ Calibration was performed at 25% power (≈5.0 mW cm^−2^), and all subsequent tests were conducted under these conditions with a 0.3% strain amplitude at 2 Hz. Oxygen depletion times were averaged over three replicate experiments.

### TVAM Printing

For the preliminary tests, a TVAM printer with a 25 mm vial was used (all printer details were provided in the recent publication).^[^ ^1^, ^12^ ^]^ The projector, a Wintech PRO6500S, employs a 405 nm light source and delivers a full light intensity of 44.5 mW cm^−2^ at the projection image plane. At the vial center, the pixel size measured 0.0486 mm. Projections were generated using a previously detailed algorithm that accounts for diffusion and the refraction at the air‐vial interface.^[^ ^1^, ^32^ ^]^

For large volume printing tests, a large volume TVAM printer was built, which was composed of a EKB DLPC900 projector focused onto a cylindrical vial mounted to a Physik Instrumente rotation stage (M‐060.PD). The pixel size at the vial center was 0.0855 mm. The optical configuration was analogous to cone‐beam computed tomography with diverging rays in both transverse directions to the optical axis. To account for diverging rays and refraction at the air‐vial interface, tomographic projections were computed using 3D ray tracing, and optimized using a proportional‐integral feedback loop or PIHE.^[^ ^4^, ^25^ ^]^ Briefly, PIHE was based upon object‐space model optimization (OSMO) wherein the target design was modified in object space such that the computed dose, when thresholded, resembles the original target design.^[^ ^23^ ^]^ Importantly, it was assumed that at a certain dose level gelation of the photoresin will occur (D_high_), but up to a certain dose the photoresin remains unaltered (D_low_). Ideally, all voxels inside the part (in‐part) should receive dose exceeding D_high_, whereas all voxels outside the part (out‐of‐part) should receive dose less than D_low_. Consequently, there exists a dose gradient where in‐part and out‐of‐part voxels meet (i.e., edge of printed part) which could lead to reduced quality at part surfaces. Prior to iterative optimization, the target geometries were filtered in object space using a 2D linear frequency ramp filter.^[^ ^23^ ^]^ In addition, due to limited computational power, a down sampling factor of 2 was applied for larger prints (typically those exceeding 40 mm in length). The optimization parameters used are listed in Table S4 (Supporting Information).

The system was programmed to synchronize projection display with rotational motion, typically operating at 40 ° s^−1^. At one projection per degree, this corresponds to a nominal display frequency of 40 Hz (0.025 s per image). The system prioritizes image display to maintain synchronization, and the average cycle time was ≈0.020 s. Printing progress, including frame count, exposure duration, and total rotations, was tracked with sub‐second precision (±0.025 s). Recorded video was time‐index matched to rotational position, and if camera acquisition lags, frames were omitted rather than compromising timing accuracy.

### Post Processing

Finished prints were carefully removed from the vial and immersed in isopropyl alcohol (IPA 98%, Sigma–Aldrich) for 5–10 min with gentle shaking with an orbital shaker to remove any uncured photoresin. The prints were then transferred to a fresh IPA bath (150 mL) with TPO‐L (0.2 g) and soaked for an additional 10–20 min under gentle agitation. Following the IPA/TPO‐L wash, the parts were air‐dried for 60 min before undergoing post‐curing for 60 min at room temperature in a Formlabs Form Cure L unit using 405 nm light. Finally, the parts were briefly submerged in an acetone bath (1–2 min) and then allowed to dry, resulting in prints that were no longer tacky.

### µCT Analysis

Printed samples were scanned with a Bruker Skyscan 1275 µCT system at pixel size of 35 µm. The resulting images were reconstructed using Bruker's Skyscan NRecon software, and signed‐distance fields were subsequently extracted from the scans with MeshLab.

### SLA Printing

A 3DBenchy model measuring 36 mm in length was printed using a Form 4 SLA printer with Formlabs Clear Resin V5. The print was executed using a layer thickness of 100 µm under default settings. Following printing, the samples were washed in IPA for 10 min and subsequently post‐cured in a Formlabs Form Cure chamber at 60 °C for 15 min, in accordance with the manufacturer's recommendations.

### DLP Printing

A 36 mm 3DBenchy was fabricated using an Asiga Pro 4K UV385 DLP printer. The optimized printing parameters included a light intensity of 17 mW cm^−2^, a slice thickness of 100 µm, an exposure time of 2 s per layer, and a burn‐in exposure of 10 s for the initial layer, with one burn‐in layer applied. These settings were determined to yield the highest print quality after iterative testing and adjustment. During printing, Formlabs Clear Resin V4 was preheated to 25 °C. Post‐processing involved washing the printed samples in IPA for 10 min followed by curing in a Formlabs Form Cure chamber at 60 °C for 15 min, as recommended by the manufacturer.

### FDM Printing

A 36 mm 3DBenchy was printed using an Original Prusa i3 MK3 printer equipped with a 0.4 mm nozzle. The model was fabricated using 0.20 mm Prusament PETG filament with a 100 % infill.

### Statistical Analysis

All quantitative data were presented as mean ± standard deviation, unless otherwise stated. For each measurement reported, a minimum of three independent samples (n = 3) were tested to calculate the average and sample standard deviation. No data transformations or normalizations were applied, and all outliers were included in the analysis unless obvious experimental error was observed.

## Conflict of Interest

The authors declare no conflict of interest.

## Supporting information



Supporting Information

Supplemental Video1

Supplemental Video2

Supplemental Video3

Supplemental Video4

Supplemental Video5

## Data Availability

The data that support the findings of this study are available from the corresponding author upon reasonable request.
