# Supplementary material for: Advancing Tomographic Volumetric Printing Via Oxygen Inhibition Control: Improved Accuracy and Large‐Volume Capability
Source: Adv Mater. 2025 Sep 12;37(47):e08729. doi: 10.1002/adma.202508729 (PMC12651127; doi:10.1002/adma.202508729)
Supplement: Supplementary file 1 — Supporting Information [file ADMA-37-e08729-s002.docx]

Supporting Information

**Advancing Tomographic Volumetric Printing via Oxygen Inhibition Control: Improved Accuracy and Large-Volume Capability**

*Yujie Zhang*, Katherine Houlahan, Daniel Webber, Nicolas Milliken, Kathleen L. Sampson, Hendrick de Haan, Hao Li, Robynne Vlaming, Liliana Gaburici, Antony Orth, Chantal Paquet**

Scheme S1. Detailed reaction mechanisms of acrylate photopolymerization including photolysis, initiation, oxygen depletion, propagation, and termination as well as detailed mechanisms of amines, thiols, and phosphines as anti-oxygen inhibition additives.


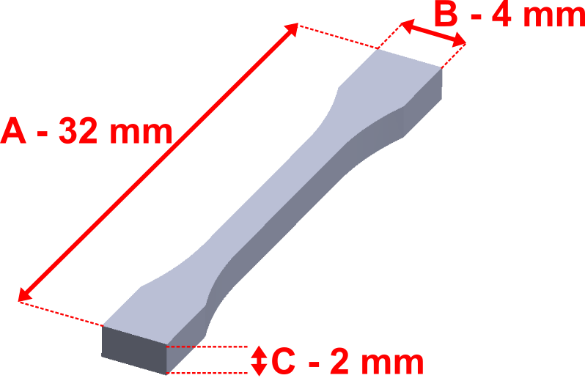


Figure S1. Dog bone print model, based on ISO-527-2 Type 1BB, for screening the three different additives and at various concentrations.


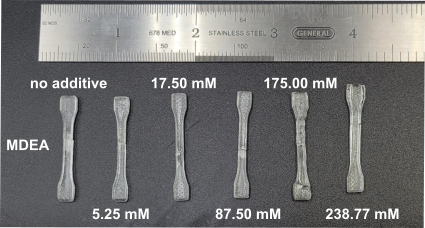


Figure S2. Image of dog bone prints with various concentrations of MDEA additive.


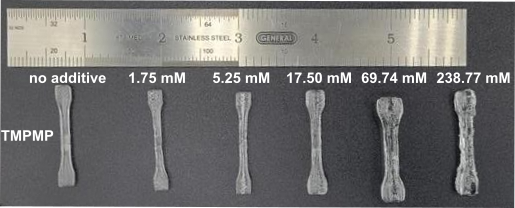


Figure S3. Image of dog bone prints with various concentrations of TMPMP additive.


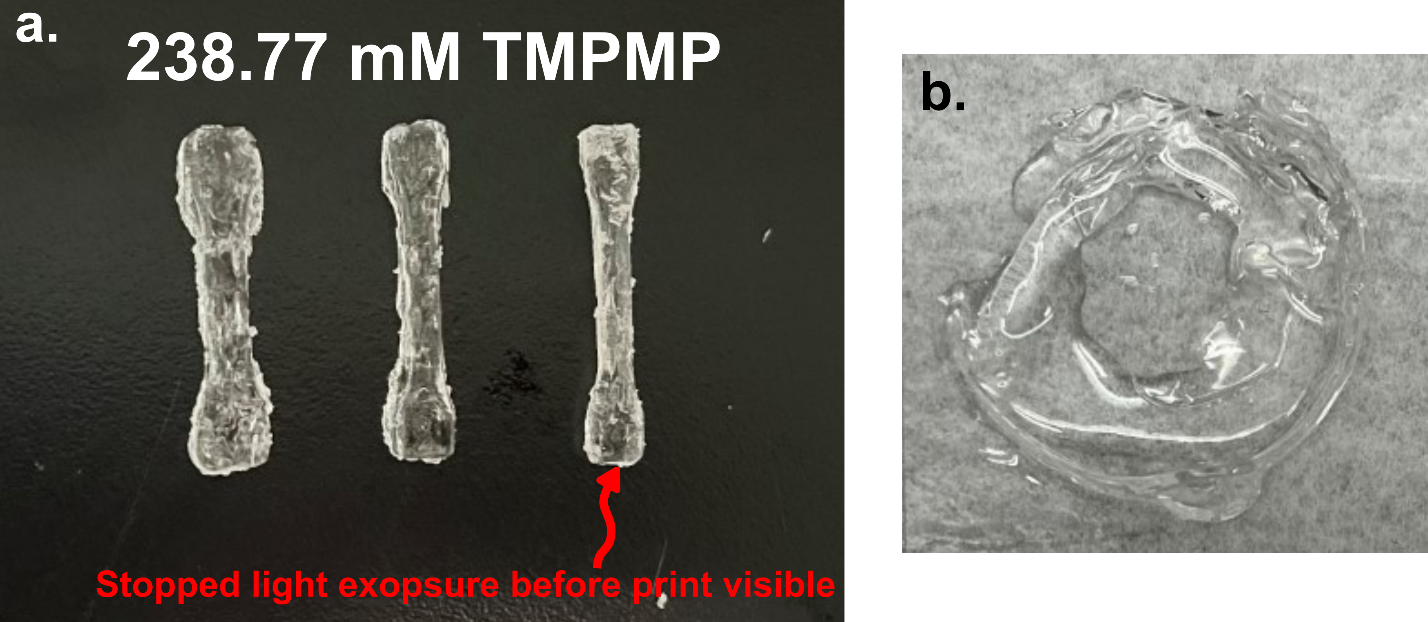


Figure S4. a) Image of TMPMP dog bones with 238.77 mм TMPMP additive with poor resolution. The light illumination for the dog bone on the far right was stopped before the print was visible during printing via our Optical Scanning Tomographic (OST) imaging method. b) Ring of polymer formed in out-of-part regions around dog bones printed with 69.74 and 238.77 mм TMPMP additive.


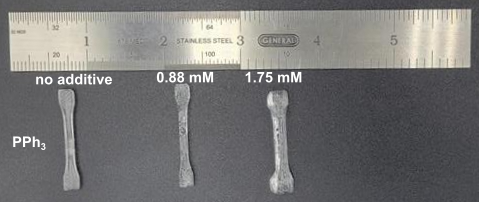


Figure S5. Image of dog bone prints with various concentrations of PPh_3_ additive.


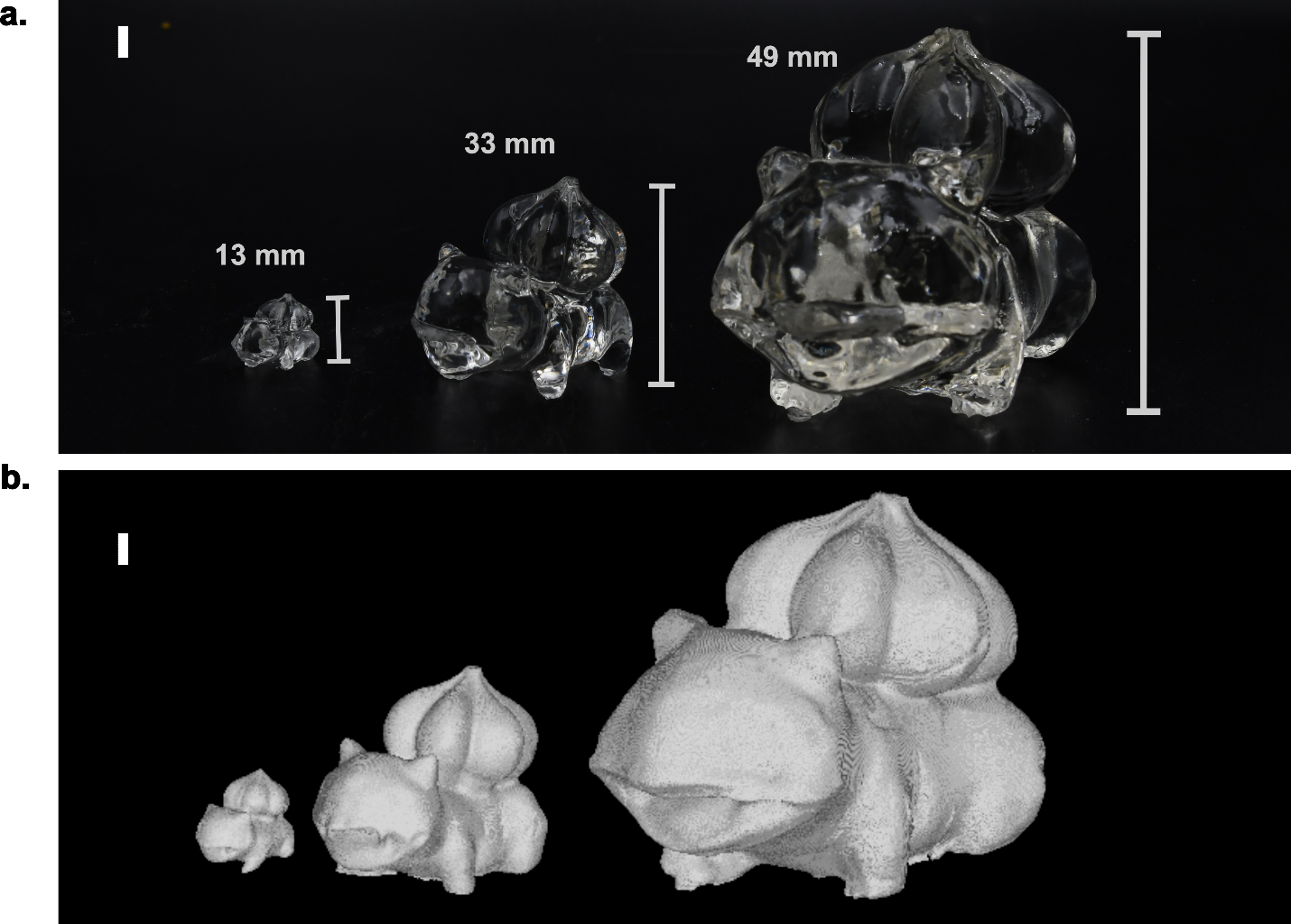


Figure S6. (a) Series of Bulbasaur prints with height of 13.0, 32.5, and 49.0 mm, illustrating the scalability of the process. (b) µCT reconstruction of the Bulbasaur shown in (a).


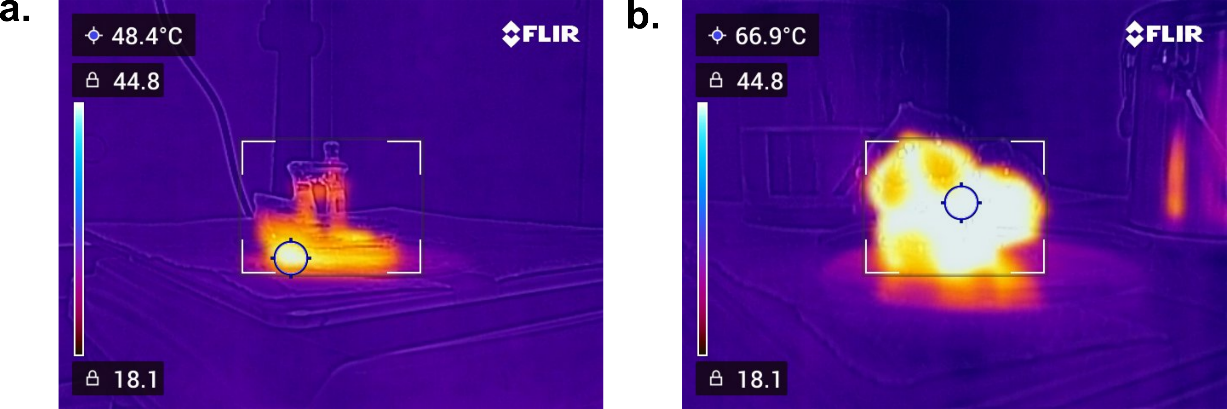


Figure S7. Heat image of a 60 mm Benchy and Bulbasaur taken after removed from photoresin.


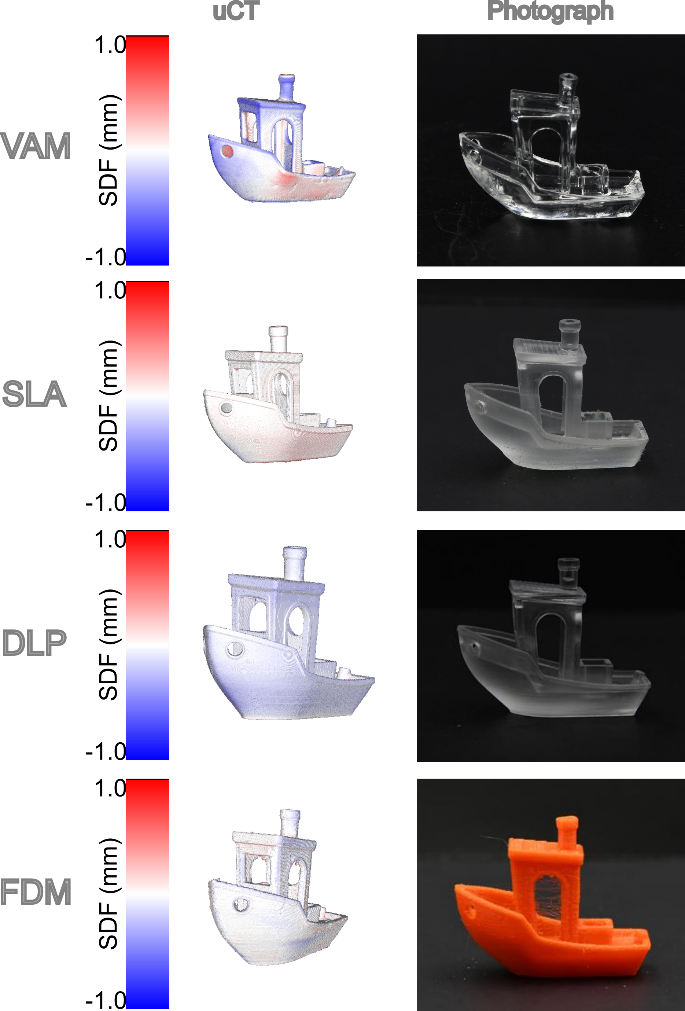


Figure S8. Comparison of print quality for a 36 mm-long 3DBenchy fabricated using different 3D printing techniques.

Table S1. Variables for allometric1 power model fits of oxygen depletion curves for each additive using Origin 2022b.

| **Figure** | **Additive** | **a** | **b** | **R-Square (COD)** |
| --- | --- | --- | --- | --- |
| **Figure 3b** | MDEA | 50.19 ± 10.21 | -0.34 ± 0.05 | 0.90 |
| **Figure 3b** | TMPMP | 44.92 ± 10.82 | -0.48 ± 0.12 | 0.90 |
| **Figure 3b** | PPh3 | 7.41 ± 0.61 | -0.55 ± 0.11 | 0.95 |
| **Figure 4a** | MDEA | 139.49 ± 6.18 | -0.40 ± 0.02 | 0.99 |

Table S2. Print fidelity of printed 3DBenchy (36 mm length) using D8P2 photoresin with 0.5 mм TPO-L at various MDEA concentrations.

| **Amine Concentration (mм)** | **Max Difference (mm)** | **Min Difference (mm)** | **Mean Difference (mm)** | **RMS (mm)** |
| --- | --- | --- | --- | --- |
| 25 | 2.234 | -2.543 | -0.073 | 0.628 |
| 50 | 2.099 | -0.978 | 0.288 | 0.507 |
| 75 | 2.100 | -0.959 | 0.285 | 0.440 |
| 100 | 1.236 | -0.982 | 0.040 | 0.256 |
| 125 | 1.025 | -0.796 | 0.027 | 0.175 |

Table S3. Fine feature resolution in 36 mm 3DBenchy prints using D8P2 photoresin with 0.5 mм TPO-L and varying MDEA concentrations. For each feature, its presence is indicated; if present, the dimensional deviation from the design (in mm) is also reported.

| **MDEA Concentration (mм)** | **Port hawsepipe (Figure 5 b.1)** | **Starboard hawsepipe (Figure 5 b.1)** | **Chimney (Figure 5 b.3)** | **Chimney Wall thickness (Figure 5 b.3)** | **Blind Hole (Figure 5 b.5)** |
| --- | --- | --- | --- | --- | --- |
| 25 | Not Present | Not Present | Not Present | Not Present | Not Present |
| 50 | Present (-0.937) | Present (-0.974) | Present (-0.413) | Filled | Not Present |
| 75 | Present (-0.685) | Present (-1.036) | Present (-0.289) | Filled | Not Present |
| 100 | Present (-0.071) | Present (-0.152) | Present (-1.05) | Present (0.921) | Not Present |
| 125 | Present (0.048) | Present (0.082) | Present (-0.345) | Present (-0.263) | Present (-0.224) |

Table S4. Projection simulation parameters of all prints with the corresponding print time using D8P2 with 0.5 mм TPO-L and 125 mм MDEA.

| **Geometry** | **D_low_** | **D_high_** | **Down Sampling** | **Vial Sze (mm)** | **Optimization** | **Print Time (s)** |
| --- | --- | --- | --- | --- | --- | --- |
| **3DBenchy**  **(12 mm length)** | 0.7 | 0.8 | 1 | 65 | PIHE | 58.2 |
| **3DBenchy**  **(24 mm length)** | 0.7 | 0.8 | 1 | 65 | PIHE | 43.9 |
| **3DBenchy**  **(36 mm length)** | 0.6 | 0.8 | 1 | 65 | Proportional‐Integral Feedback Loop | 79.3 |
| **3DBenchy**  **(36 mm length)** | 0.6 | 0.8 | 1 | 65 | PIHE | 43.7 |
| **3DBenchy**  **(48 mm length)** | 0.6 | 0.8 | 2 | 100 | PIHE | 48.8 |
| **3DBenchy**  **(60 mm length)** | 0.6 | 0.8 | 2 | 100 | PIHE | 44.1 |
| **3DBenchy**  **(12 mm length) x 23** | 0.6 | 0.8 | 1 | 65 | PIHE | 54.1 |
| **M5 Screw and nut sets** | 0.6 | 0.8 | 1 | 65 | PIHE | 47.5 |
| **Hex Nut and Screw cap sets** | 0.6 | 0.8 | 1 | 65 | PIHE | 41.7 |
| **Gyroid Lattice**  **(40 mm diamter)** | 0.6 | 0.8 | 1 | 65 | PIHE | 50.8 |
| **Kevin Lattice**  **(30 mm diameter)** | 0.6 | 0.8 | 1 | 65 | PIHE | 83.9 |
| **Bulbsaur**  **(16 mm length)** | 0.6 | 0.8 | 1 | 65 | PIHE | 41.3 |
| **Bulbsaur**  **(40 mm length)** | 0.6 | 0.8 | 1 | 65 | PIHE | 35.2 |
| **Bulbsaur**  **(60 mm length)** | 0.6 | 0.8 | 2 | 100 | PIHE | 49.7 |

Table S5. Comparison of TVAM with state-of-the-art 3D printing technologies.

| System | Printing Method | Voxel Size (µm) | Voxel Volume (mm³) | Print Volume (mm³) | Scan Speed (mm s^-1^) | Voxel Throughput  (voxel s^-1^) | Print Speed  (mm^3^ s^-1^) |
| --- | --- | --- | --- | --- | --- | --- | --- |
| Nanoscribe GT2^[1]^ | Two-photon polymerization | 0.16 × 0.16 × 1 | 2.6 × 10^-11^ | 1.0 × 10^2^ | ~100 | 6.3 × 10^5^* | 1.6 × 10^-5^ |
| Nanoscribe Quantum X (2G)^[2]^ | Two-photon polymerization + grayscale | 0.1 × 0.1 × 0.5 | 5.0 × 10^-12^ | 8.0 × 10^3^ | ~99 | 9.9 × 10^5^* | 5.0 × 10^-6^ |
| Femtika   Nanofactory^[3]^ | Multi-photon polymerization | 0.15 × 0.15 × 0.15 | 3.4 × 10^-12^ | 1.0 × 10^1^ | ~30 | 2.0 × 10^5^* | 6.8 × 10^-12^ |
| Xolography^[4]^ | Light Sheet Printing | 5.0 x 5.0 x 10.0 | 2.5 × 10^-7^ | 1.3 × 10^4^ | NA | 2.5 × 10^6^ | 5.5 × 10^1^ |
| Dynamic Interface^[5]^ | Dynamic Interface | 15.1 x 15.1 x 3** | 6.8 × 10^-5^ | 3.4 × 10^4^ | NA | 1.29 × 10^8[6]^ | 1.7 × 10^2^ |
| TVAM (25 mm Vial)^[7]^ | Tomographic Volumetric Printing | 48.6 x 48.6 x 48.6 | 1.2× 10^-4^ | 1.1 × 10^4^ | NA | (1.1 -3.3) × 10^6^ | (1.3 – 3.8) × 10^2^ |
| TVAM (100 m Vial, this work) | Tomographic Volumetric Printing | 85.5 × 85.5 × 85.5 (theoretical) / 171 × 171 × 171 (as demonstrated)*** | 6.3 × 10^-4^ (theoretical) / 5.0 × 10^-3^ (as demonstrated) | 1.8 × 10^5^ | NA | (3.2 – 9.7) × 10^6^ (theoretical) / (4.0 – 12.1) × 10^5^ (as demonstrated) | (2.0 – 6.0) × 10^3^ |

*Voxel throughput calculated by dividing the scan speed by the voxel size.^[8]^

** Layer height is calculated with a vertical speed of 150 µm s^-1^, and frequency of 50 Hz.

***As demonstrated using a downsampling factor of 2 for prints exceeding ~40 mm.

Table S6. Refractive index measurements for photoresins with and without additives at 405 nm.

| **Photoresin** | **Additive** | **Additive Concentration (mм)** | **Refractive Index** |
| --- | --- | --- | --- |
| DUDMA | none | none | 1.50437 |
|  | MDEA | 5.25 | 1.50290 |
|  |  | 17.50 | 1.50356 |
|  |  | 87.50 | 1.50403 |
|  |  | 175.00 | 1.50368 |
|  |  | 238.77 | 1.50269 |
|  | TMPMP | 1.75 | 1.49857 |
|  |  | 5.25 | 1.50367 |
|  |  | 17.50 | 1.50255 |
|  |  | 69.74 | 1.50434 |
|  |  | 238.77 | 1.50605 |
|  | PPh_3_ | 0.88 | 1.50371 |
|  |  | 1.75 | 1.50166 |
| D8P2 | none | none | 1.49966 |
|  | MDEA | 5 | 1.49956 |
|  |  | 25 | 1.49953 |
|  |  | 50 | 1.49960 |
|  |  | 75 | 1.49946 |
|  |  | 100 | 1.49949 |
|  |  | 125 | 1.49946 |

**References**

1. Nanoscribe Photonic Professional GT+ | Terrapin Works [Internet]. [cited 2025 Jul 3];Available from: https://terrapinworks.umd.edu/equipment/nanoscribe-photonic-professional-gt

2. Quantum X shape | High-resolution 3D printer [Internet]. [cited 2025 Jul 3];Available from: https://www.nanoscribe.com/en/products/quantum-x-shape/

3. Laser Nanofactory. Multiphoton Polymerization [Internet]. FEMTIKA [cited 2025 Jul 3];Available from: https://femtika.com/product/multiphoton-polimerization/

4. Regehly M, Garmshausen Y, Reuter M, König NF, Israel E, Kelly DP, et al. Xolography for linear volumetric 3D printing. Nature 2020;588(7839):620–4. doi: 10.1038/s41586-020-3029-7.

5. Vidler C, Halwes M, Kolesnik K, Segeritz P, Mail M, Barlow AJ, et al. Dynamic interface printing. Nature 2024;634(8036):1096–102. doi: 10.1038/s41586-024-08077-6.

6. Comparing 3D Printers [Internet]. Available from: https://3dprintingspeed.aph.kit.edu/

7. Zhang Y, de Haan H, Houlahan K, Sampson KL, Webber D, Orth A, et al. Impact of oxygen inhibition on (meth)acrylate photopolymerization in tomographic volumetric printing. Addit Manuf 2025;109:104844. doi: 10.1016/j.addma.2025.104844.

8. Hahn V, Kiefer P, Frenzel T, Qu J, Blasco E, Barner-Kowollik C, et al. Rapid Assembly of Small Materials Building Blocks (Voxels) into Large Functional 3D Metamaterials. Adv Funct Mater 2020;30(26):1907795. doi: 10.1002/adfm.201907795.
