# Supplementary figures and images for: Advancing Tomographic Volumetric Printing Via Oxygen Inhibition Control: Improved Accuracy and Large‐Volume Capability
Source: Adv Mater. 2025 Sep 12;37(47):e08729. doi: 10.1002/adma.202508729 (PMC12651127; doi:10.1002/adma.202508729)

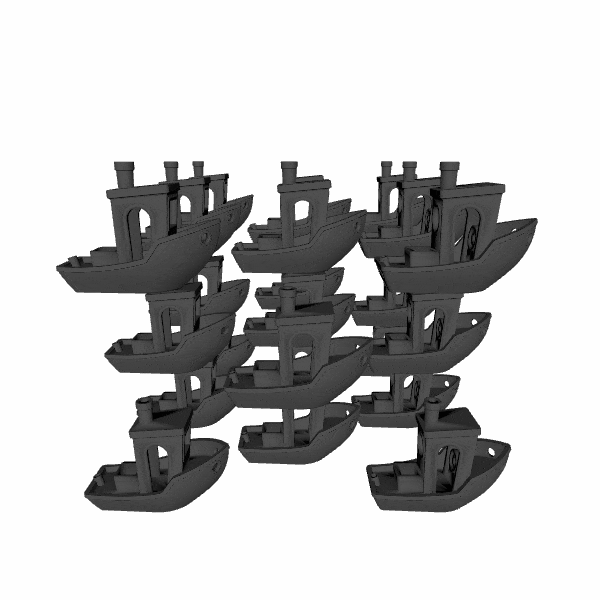

Supplement: Supplementary file 4 — Supplemental Video3 [file ADMA-37-e08729-s006.gif]

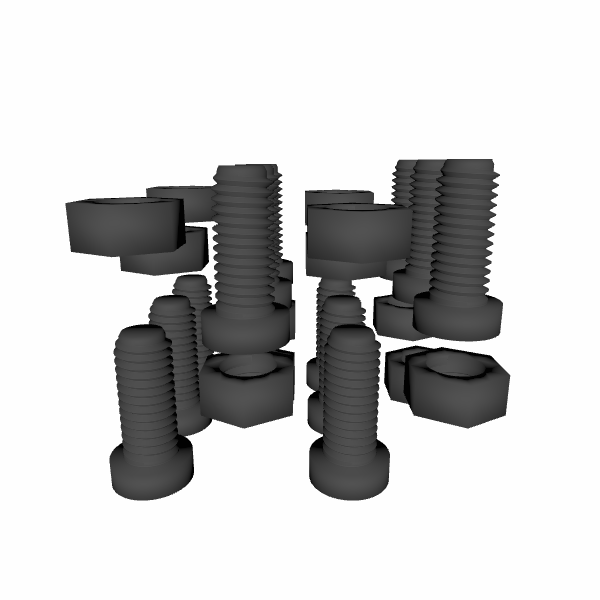

Supplement: Supplementary file 5 — Supplemental Video4 [file ADMA-37-e08729-s001.gif]

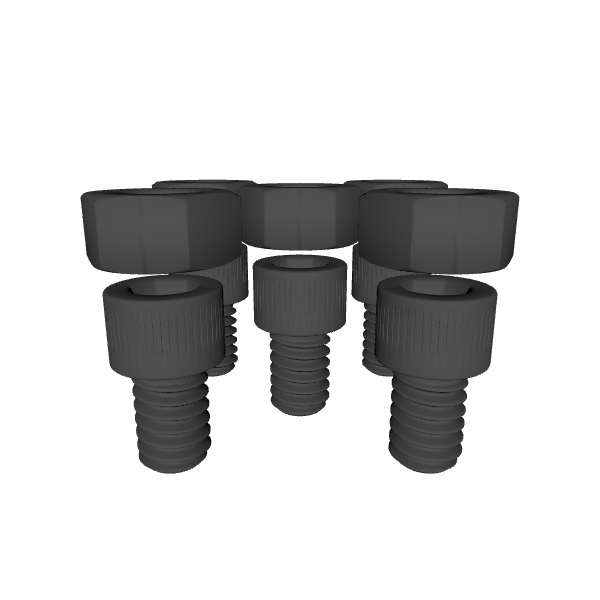

Supplement: Supplementary file 6 — Supplemental Video5 [file ADMA-37-e08729-s005.gif]
